# Atlas in progress – health care for patients with chronic illness

**DOI:** 10.1007/s43999-022-00011-5

**Published:** 2022-10-28

**Authors:** Hanne Sigrun Byhring, Barthold Vonen

**Affiliations:** Center for Clinical Documentation and Evaluation, Northern Norway Regional Health Authority, Tromsø, Norway

**Keywords:** Health service research, Variation in health care, Health atlas

## Abstract

Over the last 7 years the Norwegian health atlas service has produced a total of 11 atlases covering subjects such as psychiatric health services, gynecology, orthopedics, specialist health services for children and for the elderly and healthcare quality to mention some. The form of all 11 atlases has been more or less the same since the first atlas was published in 2015. Over time we have realized that our atlas “products” could and should be more easily accessible, to better serve as a tool for improving health care services. Therefore, as part of the process of creating our latest atlas, the *Atlas of health care for patients with chronic illness,* we have set about reinventing the visual expression of the net-based presentation. The *Atlas of health care for patients with chronic illness* also provides the first opportunity to explore the possibilities and limitations of a unique new data set covering most government-funded health care in Norway for a time period of 4 years, where we can follow each patient across levels of care. Since the publication of the first health atlas for Norway in 2015, much attention has been given to geographic variation in the use of health services. However, as far as we can see little has changed – the atlases have sparked discussions, but not as much action from the decisionmakers. It is clear to us that to spark real change, we must engage more and better with the intended users of the atlases – leaders and decisionmakers, health care professionals and patients and indeed the national ministry of health. Finding ways to achieve this will be a main focus for our efforts in the coming years.

## The Norwegian health atlas – an integral part of the specialist health service

The overriding goals of Norwegian health care service is high quality health care and equitable distribution across geography, ethnicity and socioeconomic variations. The main provider is the government with an in principle free-for all service. Private providers are available in the market place but the vast majority of services are publicly provided. Typically, most health care services from private providers can be classified as supply sensitive services (supply sensitive as defined by Wennberg [4]). All hospitals admitting patients in need of acute care are publicly owned, with a public funding based 50% on the catchment population and the services offered and 50% on a DRG based refund for services provided.

The publication of the first Norwegian health atlas – the Day Surgery Atlas – in 2015, sparked an interest in the concept of geographic variation within both the health service itself and in government agencies. This has given rise to numerous investigations in addition to, and in supplent of, the following 10 health atlases. The Norwegian population is in many aspects more homogenous than in other countries. Despite areas with rather large immigrant populations, there is a fair consensus that only a few conditions show a geographical variation in incidence and prevalence.

The health atlas service is organizationally deeply embedded in the specialist health service, and is committed to serving the needs of policy- and decisionmakers and health care professionals working towards the goal of providing better and more equal health services to the Norwegian population. We also recognize the interdependence between the atlas service and health care professionals in the process of generating change, and have adopted a model for atlas development that involves and engages health care professionals in the process of design, development and quality assurance of each atlas.

## Reinventing the Norwegian health atlas

Since the publication of the first health atlas for Norway in 2015, much attention has been given to geographic variation in the use of health services. However, as far as we can see little has changed – the atlases have sparked discussions, but not as much action from the decisionmakers. We believe that being an integral part of the specialist health service, the health atlas service has an obligation to contribute towards putting our knowledge and results to work in creating better services for the patients. When publishing new results that document high and unwarranted geographic variation, questions invariably rise as to the cause of the variation and what should be done to reduce it. So far, the health atlas service has seldom offered good answers to these questions.

We have concluded that to meet our responsibilities to the patients, it is not enough to document and spread information about variation in health care – we must also strive to answer these questions of why variation occurs and what can be done about it. To do this we need to ask new questions and employ different methods of analysis, and we need access to more data. In the last few years we have built a research group focusing on understanding the mechanisms causing variation in health care. In the coming years we will try to utilize insights and knowledge gained through research to increase the relevance and usefulness of the atlases.

In addition, we believe that the way we communicate our results can have a large impact on how and to what degree they are employed in the service of the patients. We realize that our efforts so far have not been sufficient. To engage more and better with the intended users of the atlases, we need to modernize the visual expression of the atlases, offer more updated analyses and communicate more strategically by building relations with key decisionmakers. We believe that for efforts towards changing practices to be successful, early involvement by health care professionals is key. In the future, we will increase our efforts to reach out to both health care professionals and leaders – not only with a view to communicating results, but with the aim of securing their help for increasing the relevance of our atlas products.

In total, the changes that we believe are necessary amount to nothing short of a reinvention of the Norwegian health atlas. In the following, we will describe the first steps we have made towards this goal – as part of the process of creating a new atlas; the *Atlas of health care for patients with chronic illness.*

### Choose your graph

Over the last 7 years the Norwegian health atlas service has produced a total of 11 atlases covering subjects such as psychiatric health services, gynecology, orthopedics, specialist health services for children and for the elderly and healthcare quality to mention some. The form of all 11 atlases has been more or less the same since the first atlas was published in 2015; an interactive map, thematic summaries of main findings and the main product a rather voluminous report detailing all the findings and methods applied. All these atlases are published in English (see https://www.skde.no/helseatlas/en).

Over time we have realized that our atlas “products” could and should be more easily accessible, to better serve as a tool for improving health care services. Therefore, as part of the process of creating our latest atlas, the *Atlas of health care for patients with chronic illness,* we have set about redesigning the visual expression of the net-based presentation. The *Atlas of health care for patients with chronic illness* is designed for the web, not for print, with an interactive interface allowing users to engage with and explore the material in new ways. The report is replaced by a web page designed for user-interaction. By allowing the user to choose the graph, we can allow more data to be seen from several different perspectives and at the same time communicate our results in a simple and easy to grasp model.

Further along, the interactive maps created for each atlas will be replaced with a new tool that makes it easier for the user to benchmark results for one region across one or several health care atlases, to find areas where there might be a cause for improvement in the services.

To succeed in this, while retaining the reputation of the health atlas service as a trustworthy and well-documented source of information, will require a gradual approach and courage to experiment with both written texts and tools of data visualization. We expect this to be an ongoing project of innovation and renewal over the next few years.

### Expanded access to data

The main data source for the health atlas service has been the Norwegian patient registry (NPR), which contains administrative data, demographic information and coded medical information for inpatient and outpatient treatments in the Norwegian specialist health services [2]. This registry contains information about the use of most publicly funded specialist health services (with the exception of some diagnostic services such as medical imaging) for the entire population of Norway – with high completeness and mainly of good quality – and is by itself an invaluable tool for documenting regional variation in the use of health services. Lately, the data source for the Norwegian health atlas service has been considerably expanded to include data from the Norwegian Registry for Primary Health Care (NRPHC).

From now on we have access to registry data that enables combining data from most government-funded health care in Norway for a time period of 4 years, and we can follow each patient across both levels of care. The *Atlas of health care for patients with chronic illness* provides the first opportunity to explore the possibilities and limitations of this unique data set for studies of regional variation in the use of health care. This will allow us to fill in the blanks by investigating geographic variation in the use of both primary and specialist care, which is particularly important for patients with chronic illness.

### More and more data

As the next step towards reinventing the Norwegian health atlas, we are in the process of obtaining access to yet another valuable source of data that will allow us to explore variation in the use of health care in Norway from a new perspective. By 2023, we hope to gain access to information about socioeconomic status (i.e. data on income, education, family etc.) and country of origin for all the patients included in our two current data sources, the NPR and the NRPCH. This will allow for follow-up studies, in-depth analysis and scientific research that deepens our understanding of the causes and consequences of geographic variation in the use of health services.

By combining knowledge about geographic variation in the use of health care with information about how the use of health care is affected by socioeconomic status and country of origin we hope to build a powerful tool for facilitating change towards a more equal distribution of health care in Norway.

## Better health care for patients with chronic illness

The primary goal for The Centre for clinical documentation and evaluation (SKDE) in creating our latest atlas, the *Atlas of health care for patients with chronic illness,* is to provide a starting point for leaders and health care professionals in the Norwegian specialist health service who wish to improve care for this patient group. Most chronic illnesses have a significant impact on quality of life. Patients with chronic illnesses make up a large proportion of patients treated by the specialist health services. A better understanding of regional differences in how patients with chronic illness are treated is therefore important both from the patient perspective and the health care professionals providing treatment. We hope that the *Atlas of health care for patients with chronic illness* will be a useful tool for providing better and more equal health services to patients, regardless of where they live.

### Meet the patients

In the first phase of the project, different definitions of the group of chronically ill patients were identified in the literature and explored as far as possible in the data available at the time – data from the Norwegian patient registry (NPR). In internationally published literature, there are many different definitions of chronically ill (e.g. [1, 3]). Depending on which definition is chosen, the chronically ill will include a varying proportion of patients treated in the specialist health service. A broad definition could include as much as 50–60% of all patients who are treated annually in the Norwegian specialist health service. We therefore quickly recognized the need for a set of criteria by which we could choose a subset of chronic illnesses to cover in the atlas. The criteria we identified as relevant to the project were:

1. Non-reversibility: the patient can be treated, but not cured
2. Reduced health status: the patient experiences a significant loss of health status
3. Specialized treatment: the patient needs treatment in the specialist health service over time
4. Data validity: we can identify the patient with reasonable accuracy in the data
5. Numbers: the number of patients treated per year is high enough to characterize the observed geographic variation as warranted or unwarranted

Based on these five criteria we identified the following 10 chronic illnesses to study in the atlas: psoriasis, COPD, endometriosis, epilepsy, heart failure, arthritis, inflammatory bowel disease, migraine, multiple sclerosis and Parkinson’s disease. These patient groups roughly make up 8-9% of patients who are treated annually in the Norwegian specialist health service.

The *Atlas of health care for patients with chronic illness* is published in parts. The first part, devoted to the treatment of patients with chronic neurological illnesses (MS, Parkinson, Migraine and Epilepsy) was published in april 2022.

### Finding the “right” patients

A person who has been to see a general practitioner or a specialist in an outpatient clinic, and who was registered at the time with a diagnose of e.g. epilepsy does not necessarily have epilepsy. To take account of “noise” in the data in the form of errors, preliminary diagnoses and so on, we chose to limit our patient population to those who have had at least three contacts on three separate days in the course of a four-year period from 2018 to 2021. By doing this we hope to increase data validity at the expense of excluding some patients from the analyses.

In the case of Parkinsons disease and multiple sclerosis this does not significantly reduce the patient population relative to the “base population” – i.e. all patients who had one contact or more with a relevant diagnosis in the four-year period. However, in the case of migraine the patient population in the atlas is reduced by 33% relative to the “base population” as a result of this criteria.

### Main findings so far

The six most important findings for part one of the *Atlas of health care for patients with chronic illness* are

1. The proportion of patients with multiple sclerosis who were being treated with high-efficacy drugs varied widely between the referral areas. The variation is considered unwarranted.
2. There was high geographical variation in the use of botulinum toxin for patients with migraine. Health North and Health West both had the lowest proportion of patients treated with botulinum toxin, and the lowest proportion of patients treated with CGRP inhibitors.
3. Nationally, 20% of the migraine patients are on sick leave every year. The migraine patients have an average of three sick leaves per year due to migraine.
4. Around 11,600 patients were treated annually for Parkinson’s disease in the general practitioner or specialist healthcare service. This number is higher than earlier estimates.
5. There was high geographical variation in the use of specialist consultations for patients with Parkinson’s disease.
6. Use of specialist consultations for the elderly (aged 65 and over) with epilepsy varied widely between referral areas. The variation is considered unwarranted.

An English translation is available at https://www.skde.no/helseatlas/en/v2/kronikere/.

## Concluding remarks

As an integral part of the specialist health service, the health atlas service has an obligation to contribute towards putting our knowledge and results to work in creating better services for the patients. To meet this obligation, we aim to develop our analyses by asking different questions, employing a wider range of methods and accessing more data to answer questions of why variation occurs and what can be done to reduce it. We also aim to communicate better and more strategically with the intended users of the atlases to ensure that our product answers their need. As part of the process of creating the *Atlas of health care for patients with chronic illness* we have taken the first steps towards these goals by expanding the data source to include data from primary health care as well as changing the format and visual expression of the atlas.

## Data Availability

Not applicable.

## References

[1] Amaia Calderón-Larrañaga DL-F-S-K (2017) Assessing and measuring chronic multimorbidity in the older population: A proposal for its operationalization. J Gerontol A Biol Sci Med Sci 72:1417–1423 28003375 10.1093/gerona/glw233 PMC5861938

[2] Inger Johanne Bakken AM (2019) The Norwegian patient registry and the Norwegian registry for primary health care: research potential of two nationwide health-care registries. Scand J Public Helath 48:49–55 10.1177/1403494819859737 31288711

[3] Marcello Tonelli NW (2015) Methods for identifying 30 chronic conditions: application to administrative data. BMC Med Inform Decis Mak 15:31 25886580 10.1186/s12911-015-0155-5 PMC4415341

[4] Wennberg J (2010) Tracking medicine: A Researcher's quest to understand health care. Oxford University Press, New York

